# Psychological Well-Being Among Informal Caregivers in the Canadian Longitudinal Study on Aging: Why the Location of Care Matters

**DOI:** 10.1093/geronb/gbaa159

**Published:** 2020-09-09

**Authors:** Yeonjung Lee, Alex Bierman, Margaret Penning

**Affiliations:** 1 Faculty of Social Work, University of Calgary, Alberta, Canada; 2 Department of Sociology, University of Calgary, Alberta, Canada; 3 Department of Sociology, University of Victoria, British Columbia, Canada

**Keywords:** CLSA, Depression, Life satisfaction, Positive aspects of caregiving, Stress process model

## Abstract

**Objectives:**

A caregiving stress perspective suggests that caregiving harms psychological well-being in informal caregivers, whereas a caregiving rewards perspective suggests that provision of care benefits psychological well-being. This research examines whether both perspectives apply to caregiving experiences, but differently by the primary location of caregiving (i.e., in-home, other residence, and institution), as well as by gender.

**Methods:**

We analyzed depression and life satisfaction in the nationally representative Canadian Longitudinal Study on Aging (*N* = 48,648), first comparing noncaregivers (*N* = 27,699) to a combined caregiver group (*N* = 20,949) and then stratifying caregivers by the primary location of care.

**Results:**

When considered as a single group, caregivers suffered relative to noncaregivers in terms of life satisfaction and depression. When stratified by the location of care, only in-home caregivers reported both greater depression and lower life satisfaction. Nonresidential caregivers did not differ significantly in levels of depression from noncaregivers and reported higher life satisfaction. Institutional caregivers reported greater depression than noncaregivers, but did not differ significantly in life satisfaction. These patterns were stronger among women than men.

**Discussion:**

Both the caregiving stress and caregiving rewards perspectives are applicable to the caregiving experience, with the stress perspective more applicable to in-home caregivers and the rewards perspective more relevant to nonresidential caregivers. Recommendations include targeted practice focused on the location of care as well as the gender of the caregiver. Given that nonresidential caregivers actually benefit from providing care, interventions need to focus on identifying and bolstering positive aspects of the caregiving experience.

This study examines psychological well-being among individuals who care for someone, with caregiving necessitated by a health condition or limitation. Caregiving involves a number of demands that can increase stress (Lawton et al., 1991; Pearlin et al., 1990). Evidence supporting the pernicious consequences of stress for psychological well-being suggests that caregivers may be at increased risk for deteriorated psychological well-being (Pearlin & Bierman, 2013). Yet, providing care for others also includes personal rewards that can enhance psychological well-being through the provision of a sense of fulfillment and meaning (Folkman, 2001). For example, caregiving may be associated with growth and well-being (Jones et al., 2011). Furthermore, although research documents that caregivers endure considerable challenges (Pinquart & Sörensen, 2003), positive outcomes for caregivers, such as feeling rewarded and satisfied, have also been observed (Greenwood et al., 2008; Pierce et al., 2007). Therefore, it has been suggested that we need to avoid an exclusively “dismal” view of caregiving and attend to both negative *and* positive outcomes (Lynch et al., 2018, p. 1).

One key to reconciling caregiving stress and caregiving rewards perspectives may be through a focus on the caregiving context such as the location of caregiving. The caregiving context usually references factors such as geographic setting, community resources, living situation/proximity, and financial resources and characteristics (Young et al., 2020). Our study focuses on the primary location of care, based on the view that “(t)he site where care occurs influences how care practices are shaped and experienced …” (Torgé, 2020, p. 378). Previous studies have examined the role of living arrangements in caregiving, but most studies do not differentiate among the implications of caring for a person living in the same household (in-home/coresidence), caring for someone living in another household (outside residence/nonresidential), and caring for someone in an institutional facility. Research suggests that caregivers are at the greatest risk of negative psychological outcomes when they live with the care recipient in the same household (National Academies of Sciences, 2016), but the relevance of the location of care for positive outcomes or the potential to derive benefits from the caregiving experience is not clearly indicated in previous literature (Brown & Brown, 2014; Roth et al., 2015).

In addition, gender forms a critical context for caregiving experiences (Revenson et al., 2016), as it is well established that women are more vulnerable than men to caregiving stress, as women who are caregivers tend to report a greater burden of care and have less access to coping resources than men who are caregivers (Swinkels et al., 2019). Less clear, however, is whether the same gender difference applies to the case of rewarding and satisfying outcomes, as well as how gender moderates the association between the location of care and psychological well-being. Previous studies of caregivers have often used samples that are largely or completely composed of women, thereby limiting the opportunity for gender comparisons (Revenson et al., 2016).

To address these gaps, we examine whether caregiving is differentially associated with negative and positive aspects of psychological well-being depending on the location of care. The following research questions are addressed: (a) How is the location of care associated with caregivers’ depressive symptoms and life satisfaction? (b) Does the relationship between the location where care is provided and caregivers’ psychological well-being differ between women and men? By answering these questions and by focusing on the entire population instead of caregivers only, thereby allowing for comparisons with noncaregivers (Hango, 2020), this research will test whether the location of care is a critical contingency when it comes to the psychological well-being implications of informal caregiving.

## Multiple Aspects of Caregivers’ Well-Being and the Location of Care

This study is framed by the stress process (Pearlin & Bierman, 2013) and caregiver empowerment models (Jones et al., 2011). The stress process model (SPM) is a sociologically oriented model that underscores the degree to which individual placement in structures of social stratification condition the causes and consequences of stress exposure for well-being (Pearlin & Bierman, 2013). The model emphasizes that disadvantaged social statuses can accentuate the effects of stress by intensifying the extent to which stress exposure leads to additional stressors, as well as by limiting access to psychosocial resources and the coping efficacy of these resources (Pearlin, 1999). When applied specifically to caregiving, the SPM integrates the characteristics of the caregiving situation as an additional critical factor determining the psychological consequences of caregiving. The model suggests that characteristics of the caregiver and the situation intersect to form a context that shapes the stress process, with caregiving demands viewed as stressors that influence individuals’ health and well-being (Pearlin et al., 1990).

Whereas the SPM focuses attention on the negative implications of caregiving, the caregiver empowerment model (CEM) provides a framework for understanding the positive outcomes that may also result from caregiving. According to the CEM, factors that influence the process of empowerment include background characteristics (demographics, acculturation, and prior relationships), caregiving demands (care receiver impairment, caregiving activities, and competing role demands), filial values (responsibility, respect, care), personal/family/community resources, appraisals of challenge or stress, and caregiving outcomes. For example, this model notes that family and community resources “can empower the caregiver to manage the situation effectively and achieve positive health outcomes” (Jones et al., 2011, p. 14). The CEM, therefore, suggests not only that negative outcomes can be avoided but also, that with sufficient support resources, the caregiver is likely to perceive caregiving demands as meaningful challenges, with consequent positive effects.

Previous caregiving research has focused heavily on the adverse outcomes of caregiving. Research shows that when caregivers are compared to noncaregivers, caregivers report higher levels of stress and more symptoms of depression (Pinquart & Sörensen, 2003). Research has also examined positive outcomes, supporting the CEM by demonstrating that caregivers also describe their experiences as satisfying and rewarding (Greenwood et al., 2008; Pierce et al., 2007). A systematic review explored how positive aspects of caregiving affect the well-being of caregivers and reported positive aspect of caregiving is positively associated with global measures of well-being such as satisfaction with life and quality of life (Quinn & Toms, 2019). The review also identified that some studies employed life satisfaction as a measure of well-being for caregivers, which coheres with conceptual overviews that conclude that life satisfaction provides distinct information from measures of negative emotions on individual understandings of well-being (Pavot & Diener, 2008), as well as a body of empirical work demonstrating the sensitivity of measures of life satisfaction to variations in life circumstances (Diener et al., 2013). Although additional evidence suggests the coexistence of caregiving burdens and satisfaction among caregivers (Andrén & Elmståhl, 2005), studies have tended to focus on one or the other without considering that caregiving outcomes could be both positive and negative simultaneously. In part, then, both the SPM and CEM may be relevant to the caregiving experience, as caregivers may experience negative effects from caregiving stress while also experiencing benefits in terms of satisfaction from the provision of care. In this research, we therefore examine both the negative psychological outcome of depression and the positive outcome of life satisfaction.

Although both the SPM and the CEM may be relevant to caregiver outcomes, they likely differ in their relevance based on the location in which care takes place. Some research shows that coresiding with the care recipient can have a detrimental impact on a caregiver’s psychological well-being due to the increased demands of caregiving (Arai et al., 2014; Mentzakis et al., 2009). Caregiving stress may also be linked to coresidence because coresidential caregivers might be unable to obtain any respite from caregiving, and respite can be critical for caregiver functioning (Evans, 2013). However, caregivers who do not share a residence with the care receiver may experience feelings of guilt and fear that they are not doing enough. Another stream of evidence suggests that, when compared with those who do not coreside with the care receiver, coresidential caregivers can show lower levels of burden and depressive symptoms (Ayalon & Green, 2015; Li et al., 2019). Other findings indicate that coresidential caregivers report both an increased sense of gain and strain from the caregiving experience (Berg-Weger et al., 2000), thus suggesting that “living with the care-recipient elicits both a positive and negative response from the caregiver” (p. 57).

When it comes to studying caregivers of individuals residing in institutional settings, studies report finding that caregivers’ reports of burden and strain decrease significantly after institutionalization (Gaugler et al., 2009; Kong, 2008), suggesting that institutional caregivers may well have better psychological well-being than in-home caregivers and/or caregivers providing care outside of their own residence (hereafter referred to as nonresidential caregivers) due to the reduced demands associated with caregiving. On the other hand, researchers also note that, generally, caregiving does not end following institutionalization (Davies & Nolan, 2006; Stull et al., 1997). Findings indicate that family caregivers tend to report being depressed following the institutionalization of their relatives and that depression levels do not decrease significantly following institutionalization (Kong, 2008). In fact, some studies comparing in-home and nursing home caregivers report finding higher rather than lower levels of stress among nursing home caregivers (Stephens et al., 1991), with others reporting no difference in stress levels (Dellasega, 1991) or depression (Lieberman & Fisher, 2001; Stephens et al., 1991) between the two groups. This study contributes to the literature by shedding additional light on these mixed findings by comparing caregiving in three different care locations (i.e., in-home, other residence, and institution) to noncaregivers.

## Location of Care and Caregivers’ Gender

The social characteristics of the caregiver can shape the ramifications of caregiving for well-being. Key among these characteristics is the gender of the caregiver. The extensive body of evidence indicating gender differences across caregiving experiences and outcomes renders a failure to consider gender in the study of caregiving “short sighted” (Revenson et al., 2016, p. 49). Several studies suggest that the nature of caregiving and its effects on caregiver well-being differ for men and women (Pinquart & Sörensen, 2006). In general, women caregivers experience greater depression in conjunction with caregiving than men caregivers (McDonnell & Ryan, 2013; Pinquart & Sörensen, 2006). Less well understood, however, is whether there is a gender difference in regard to the experience of caregiving as satisfying and rewarding. Even less is known regarding whether the impact of the location of care on caregivers’ psychological well-being differs by gender.

Some explanations for gender differences in the consequences of caregiving are that they reflect differences in caregiving stressors, such as the intensity and type of care provided (Revenson et al., 2016). For example, women typically provide more intensive personal care and longer hours of care than men (Pinquart & Sörensen, 2006). Gender differences in the impact of caregiving on employment are also important, as caregiving is associated with decreased involvement in the labor force among women but not men (Lee et al., 2015). Given that some caregivers consider their workplace a respite from the demands of caregiving (Carmichael & Charles, 2003), lack of employment might also enhance the stress of caregiving demands. If so, caregiving stress is likely to be greater for women than for men, and as a result, the adverse psychological well-being effects of caregiving might be stronger for women than for men (Uccheddu et al., 2019).

The psychological rewards of caregiving may also vary by gender, with the disproportionate stresses of caregiving among women outweighing the benefits as well as with the result that the positive effects of caregiving are evident more strongly in men. Yet, it is rare that both positive and negative outcomes of caregiving have been examined in tandem when gender differences in the consequences of caregiving are considered. Given the dearth of research that compares the applicability of the SPM and CEM perspectives to men and women, as well as little attention to the intersection of gender and the location of care, we address this gap by examining whether the relevance of location of care for depression and life satisfaction in turn differs between women and men.

## Method

### Data

This study draws on the Canadian Longitudinal Study on Aging (CLSA). The CLSA baseline sample includes 51,338 respondents aged 45–85 years and the survey combines data obtained from two sub-surveys—a tracking cohort survey and a comprehensive cohort survey. Tracking cohort respondents (*N* = 21,241) were randomly selected within age/sex strata for each province and were interviewed by telephone. They were recruited in three ways: (a) from a previous large-scale social survey (the Canadian Community Health Survey on Healthy Aging), (b) through mail-outs from provincial health ministries, and (c) by means of random-digit dialing. Comprehensive cohort respondents (*N* = 30,097) were also randomly selected based on age/sex strata, but all strata were between 25 and 50 km of one of 11 data collection sites across the country (located in Surrey, Victoria, Vancouver, Calgary, Winnipeg, Hamilton, Ottawa, Montreal, Sherbrooke, Halifax, and St. John’s), with distance depending on population density. Comprehensive cohort respondents received an in-home interview with questions similar to those administered to tracking cohort respondents. Further details of the survey can be found in the studies of Kirkland et al. (2015) and Raina et al. (2009). Because the measures used in this study were present in both the tracking and comprehensive components, the two subsamples are combined in all analyses. Multiple regression models control for survey cohort membership to account for differences that may reflect survey type.

### Focal Measures

#### Psychological well-being

An established body of work illustrates that negative emotions and life satisfaction represent related but distinct indicators of individual reflections of well-being (Keyes et al., 2002; Pavot & Diener, 2008). We therefore examine both symptoms of depression and life satisfaction to more fully represent underlying levels of psychological well-being than would be demonstrated through only one indicator. Symptoms of depression are measured using a 10-item version of the Center for Epidemiologic Studies-Depression scale (CES-D) that has been validated for use with older adults (Andresen et al., 1994). The CES-D includes eight negative symptoms and two positive symptoms. All responses to negative items were reverse-coded, so that higher scores indicated more frequent symptoms of depression (Cronbach’s α = 0.87). Life satisfaction is measured using the Satisfaction with Life Scale (Diener et al., 1985). The sum of responses to the five items was used to measure life satisfaction with higher scores reflecting greater life satisfaction (Cronbach’s α = 0.84).

#### Caregiving

Caregiving was measured in two different ways. Respondents were first asked if they had provided any type of assistance to another person because of a health condition or limitation in the previous 12 months. Responses to this question were coded dichotomously (0 = no caregiving and 1 = caregiving). After this, respondents were asked, “Is the person to whom you provided the most assistance…” with responses of “Living in your household,” “Living in another household,” “Living in a health care institution,” and “Now deceased.” Responses were coded into a set of dichotomous variables to measure the location of caregiving: in-home, other residence, and institution, with noncaregivers as the comparison group. Respondents who indicated that the caregiving recipient was deceased were not included in the analyses because this category likely confounded the mental health effects of caregiving and bereavement. To adjust for multiple caregiving recipients, a dichotomous variable was included in all regression analyses in which a value of 1 indicated that care was provided to two or more recipients.

*Gender* was coded so that 0 = men and 1 = women.

### Control Measures

Our analyses include controls for a number of sociodemographic, social network, and socioeconomic factors linked to caregiving and psychological well-being. We control for age in years to take into account life course differences in caregiving abilities and mental health. Differences in social network resources that may contribute to caregiving demands and psychological well-being are taken into account by controlling for marital status, a count of the number of people in a respondent’s household, the number of children, the number of siblings, and urbanicity, with urban residence compared to rural residence and rural/urban mixed residence. To control for the impact of socioeconomic differences in caregiving demands and psychological well-being, three socioeconomic indicators were included: education, total household income, and employment status. Descriptive statistics for all study variables are presented in Table 1.

**Table 1.** Study Descriptives

	Noncaregivers (55.69%)	Caregivers (44.31%)	Total sample	*p*
*Proportions*				
Women	0.476	0.551	0.509	***
Marital status				
Divorced	0.106	0.104	0.105	***
Widowed	0.072	0.053	0.064	
Never married	0.078	0.077	0.077	
Urbanicity				
Rural	0.139	0.151	0.145	**
Urban/rural mixed	0.049	0.049	0.049	
Education				
High school	0.182	0.175	0.179	***
Trades	0.120	0.110	0.116	
Non-university	0.216	0.234	0.224	
Bachelor’s degree	0.230	0.246	0.237	
Above bachelor’s	0.184	0.186	0.185	
Household income				
$20,000 to $49,999	0.207	0.188	0.199	***
$50,000 to $99,999	0.318	0.339	0.327	
$100,000 to $149,999	0.189	0.207	0.197	
$150,000 and more	0.178	0.176	0.177	
Income nonresponse	0.059	0.050	0.055	
Employment				
Full retirement	0.369	0.344	0.358	***
Partial retirement	0.092	0.105	0.098	
Unemployed	0.050	0.058	0.053	
Type of survey				
Comprehensive	0.601	0.572	0.588	***
Location of care				
In-home	—	0.230	0.102	
Other residence	—	0.664	0.294	
Institution	—	0.106	0.047	
Multiple care recipients	—	0.415	0.184	
*Means*				
Age	60.450	59.147	59.873	***
Number of people in household	2.429	2.498	2.459	***
Number of children	2.166	2.135	2.152	*
Number of siblings	2.911	2.905	2.908	

*Note: N* = 48,648. All descriptives are weighted and significance tests are based on survey estimation that takes sampling strata into account.

*p≤ .05, **p ≤ .01, ***p ≤ .001 (two-tailed tests).

### Analytic Strategy

Psychological well-being is analyzed using ordinary least squares regression. Each outcome is analyzed using two sets of models. The first set employs the dichotomous caregiving indicator as the focal predictor, and the second set uses the three locations of caregiving indicators. Each set is composed of three models: (a) a bivariate association that does not include controls, (b) a main effects model that includes all controls, and (c) an interaction model that tests whether the associations between caregiving and psychological well-being differ significantly between men and women.

All analyses are conducted using Stata 14.2. Analyses are weighted for nationally representative estimates. Variance estimation takes sampling strata into account using Stata’s survey-setting and survey-estimation commands. Deletion of recently bereaved caregivers reduced the sample size to 50,037, and listwise deletion further reduced it to 48,648 respondents. As missing responses reduced the sample by less than 3%, bias due to listwise deletion could be considered minimal.

## Results

### Differences in Psychological Well-Being Between Caregivers and Noncaregivers

Model 1 of Table 2 tests the mean difference in psychological well-being between caregivers and noncaregivers, without respect to the location of caregiving. Caregivers are significantly higher in depression. Caregivers are also lower in life satisfaction, but this difference is not significant. Model 2 shows that approximately one third of the association between caregiving and depression is eliminated through the inclusion of background controls, but caregivers remain significantly higher in depression. Furthermore, the deficit in life satisfaction among caregivers more than doubles with the introduction of controls, and the difference between caregivers and noncaregivers is now significant. Model 3 tests the extent to which these differences in turn differ by gender by testing interactions between caregiving and gender. Neither interaction is significant, indicating that when caregivers are considered as one category, differences in psychological well-being between caregivers and noncaregivers are not significantly different between men and women.

**Table 2.** Association Between Caregiving and Psychological Well-Being

	Model 1						Model 2						Model 3					
	Depression			Life satisfaction			Depression			Life satisfaction			Depression			Life satisfaction		
	*b*	*SE*	*p*	*b*	*SE*	*p*	*b*	*SE*	*p*	*b*	*SE*	*p*	*b*	*SE*	*p*	*b*	*SE*	*p*
*Focal predictors*																		
Caregiving	0.177	0.047	***	−0.089	0.065		0.113	0.053	*	−0.192	0.071	**	0.060	0.068		−0.218	0.093	*
Women							0.476	0.047	***	0.549	0.063	***	0.429	0.062	***	0.526	0.082	***
Caregiving × Women													0.104	0.091		0.052	0.121	
*Control variables*																		
Age							−0.048	0.004	***	0.051	0.005	***	−0.048	0.004	***	0.051	0.005	***
Divorced							0.689	0.087	***	−2.737	0.120	***	0.689	0.087	***	−2.737	0.120	***
Widowed							0.658	0.092	***	−1.159	0.121	***	0.661	0.092	***	−1.158	0.121	***
Never married							0.617	0.106	***	−2.083	0.146	***	0.617	0.106	***	−2.083	0.146	***
Number of people in household							−0.034	0.027		−0.036	0.036		−0.034	0.027		−0.036	0.036	
Number of children							−0.098	0.020	***	0.228	0.027	***	−0.098	0.020	***	0.228	0.027	***
Number of siblings							−0.049	0.011	***	0.089	0.014	***	−0.049	0.011	***	0.089	0.014	***
Rural							−0.305	0.067	***	0.701	0.087	***	−0.305	0.067	***	0.701	0.087	***
Urban/rural mixed							−0.213	0.107	*	0.333	0.146	*	−0.212	0.107	*	0.333	0.146	*
High school							−0.559	0.114	***	−0.015	0.146		−0.559	0.114	***	−0.015	0.146	
Trades							−0.430	0.123	***	−0.010	0.156		−0.431	0.123	***	−0.011	0.156	
Non-university							−0.662	0.114	***	0.084	0.145		−0.663	0.114	***	0.084	0.145	
Bachelor’s degree							−1.071	0.114	***	0.565	0.146	***	−1.073	0.114	***	0.564	0.146	***
Above bachelor’s							−1.107	0.117	***	0.566	0.150	***	−1.109	0.117	***	0.565	0.150	***
$20,000 to $49,999							−1.573	0.142	***	2.422	0.184	***	−1.574	0.142	***	2.421	0.184	***
$50,000 to $99,999							−2.364	0.145	***	3.800	0.187	***	−2.365	0.145	***	3.800	0.187	***
$100,000 to $149,999							−2.801	0.154	***	4.850	0.201	***	−2.801	0.154	***	4.850	0.201	***
$150,000 and more							−3.210	0.159	***	6.062	0.206	***	−3.211	0.159	***	6.062	0.206	***
Income nonresponse							−1.767	0.170	***	3.266	0.219	***	−1.767	0.170	***	3.266	0.219	***
Full retirement							0.377	0.073	***	0.492	0.095	***	0.377	0.073	***	0.492	0.095	***
Partial retirement							0.015	0.082		0.565	0.107	***	0.015	0.082		0.564	0.107	***
Unemployed							2.006	0.141	***	−2.941	0.181	***	2.007	0.141	***	−2.941	0.181	***
Comprehensive survey							−0.001	0.048		−0.585	0.064	***	−0.001	0.048		−0.585	0.064	***
Multiple care recipients							0.113	0.070		0.046	0.093		0.112	0.070		0.046	0.093	
Intercept	5.197	0.031	***	28.103	0.042	***	10.962	0.303	***	20.633	0.398	***	10.985	0.304	***	20.644	0.399	***
*R*^2^	0.0004			0.0000			0.0732			0.1287			0.0732			0.1287		

*Note:* Metric coefficients are presented and estimates are based on survey estimates that apply sampling weights and take sampling strata into account. *N* = 48,648.

*p ≤ .05, **p ≤ .01, ***p ≤ .001 (two-tailed tests).

### Differences in Psychological Well-Being by the Location of Care

**Figure 1.**
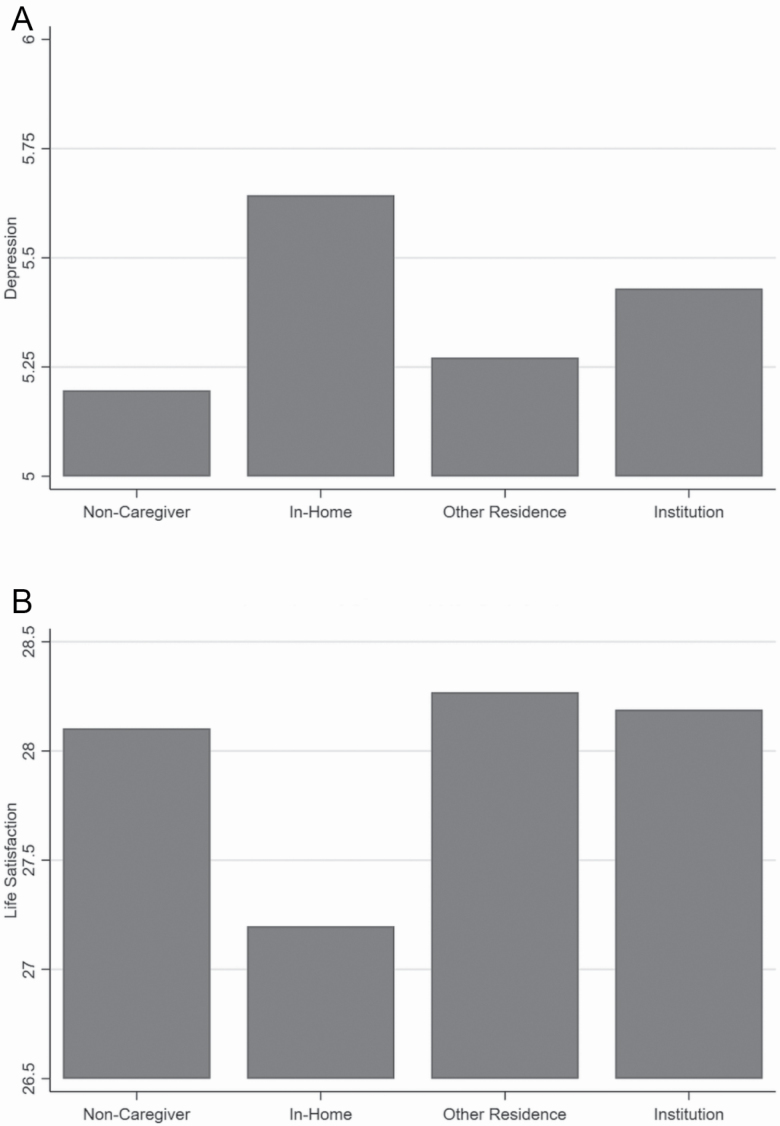
(**A**) Location of care and symptoms of depression. (**B**) Location of care and life satisfaction.

Table 3 presents a set of models in which caregivers are differentiated by the location of care and compared to noncaregivers. Model 1 tests mean differences in depression and life satisfaction, absent of any controls. Differences in psychological well-being between caregivers and noncaregivers vary significantly across locations of care, and Figure 1A and B illustrates these differences. Figure 1A shows that in-home caregivers have a substantially higher average level of depression than noncaregivers. The difference in depression between noncaregivers and nonresidential caregivers is not significant. Furthermore, although institutional caregivers have significantly higher levels of depression than noncaregivers, Figure 1A shows that this difference is much smaller than the difference between in-home caregivers and noncaregivers. Therefore, the burden of depression in caregivers is found predominantly among in-home caregivers, a pattern that is concealed when combining all caregivers into one group.

**Table 3.** Association Between Location of Caregiving and Psychological Well-Being

	Model 1						Model 2						Model 3					
	Depression			Life satisfaction			Depression			Life satisfaction			Depression			Life satisfaction		
	*b*	*SE*	*p*	*b*	*SE*	*p*	*b*	*SE*	*p*	*b*	*SE*	*p*	*b*	*SE*	*p*	*b*	*SE*	*p*
*Focal predictors*																		
In-home	0.446	0.085	***	−0.906	0.119	***	0.525	0.084	***	−1.176	0.114	***	0.254	0.107	*	−0.928	0.154	***
Other residence	0.075	0.053		0.165	0.072	*	−0.097	0.060		0.240	0.079	**	−0.076	0.079		0.148	0.106	
Institution	0.233	0.108	*	0.085	0.152		0.238	0.108	*	−0.113	0.146		0.146	0.151		−0.126	0.213	
Women							0.483	0.047	***	0.533	0.063	***	0.423	0.062	***	0.539	0.082	***
In-home × Women													0.548	0.164	**	−0.502	0.222	*
Other residence × Women													−0.020	0.102		0.154	0.134	
Institution × Women													0.184	0.210		0.018	0.286	
*Control variables*																		
Age							−0.050	0.004	***	0.056	0.005	***	−0.050	0.004	***	0.056	0.005	***
Divorced							0.710	0.087	***	−2.784	0.120	***	0.712	0.087	***	−2.786	0.120	***
Widowed							0.697	0.092	***	−1.245	0.121	***	0.705	0.093	***	−1.250	0.121	***
Never married							0.624	0.106	***	−2.099	0.145	***	0.622	0.106	***	−2.095	0.146	***
Number of people in household							−0.051	0.027		0.004	0.036		−0.052	0.027		0.005	0.036	
Number of children							−0.095	0.020	***	0.222	0.026	***	−0.095	0.020	***	0.222	0.026	***
Number of siblings							−0.048	0.011	***	0.086	0.014	***	−0.048	0.011	***	0.086	0.014	***
Rural							−0.305	0.067	***	0.703	0.087	***	−0.307	0.067	***	0.706	0.087	***
Urban/rural mixed							−0.218	0.107	*	0.344	0.145	*	−0.218	0.107	*	0.344	0.145	*
High school							−0.556	0.114	***	−0.024	0.146		−0.554	0.114	***	−0.026	0.146	
Trades							−0.424	0.123	**	−0.021	0.156		−0.425	0.123	**	−0.022	0.156	
Non-university							−0.661	0.114	***	0.082	0.145		−0.662	0.114	***	0.081	0.145	
Bachelor’s degree							−1.067	0.114	***	0.552	0.145	***	−1.066	0.114	***	0.551	0.145	***
Above bachelor’s							−1.108	0.117	***	0.568	0.150	***	−1.107	0.117	***	0.564	0.150	***
$20,000 to $49,999							−1.584	0.142	***	2.450	0.184	***	−1.587	0.142	***	2.451	0.184	***
$50,000 to $99,999							−2.374	0.145	***	3.820	0.187	***	−2.376	0.145	***	3.821	0.187	***
$100,000 to $149,999							−2.803	0.154	***	4.850	0.200	***	−2.804	0.154	***	4.851	0.200	***
$150,000 and more							−3.202	0.159	***	6.038	0.206	***	−3.205	0.159	***	6.041	0.206	***
Income nonresponse							−1.774	0.170	***	3.278	0.219	***	−1.774	0.170	***	3.277	0.219	***
Full retirement							0.383	0.072	***	0.477	0.095	***	0.380	0.073	***	0.480	0.095	***
Partial retirement							0.020	0.082		0.550	0.106	***	0.018	0.082		0.551	0.106	***
Unemployed							1.994	0.141	***	−2.910	0.181	***	1.992	0.141	***	−2.907	0.181	***
Comprehensive survey							−0.008	0.048		−0.569	0.064	***	−0.009	0.048		−0.568	0.064	***
Multiple care recipients							0.185	0.071	**	−0.111	0.094		0.178	0.071	*	−0.103	0.094	
Intercept	5.197	0.031	***	28.103	0.042	***	11.131	0.304	***	20.250	0.399	***	11.158	0.305	***	20.250	0.400	***
*R*^2^	0.0008			0.0022			0.0745			0.1322			0.0748			0.1324		

*Note:* Metric coefficients are presented and estimates are based on survey estimates that apply sampling weights and take sampling strata into account. *N* = 48,648.

*p ≤ .05, **p ≤ .01, ***p ≤ .001 (two-tailed tests).

An even starker pattern emerges for life satisfaction, as shown in Figure 1B. In-home caregivers reported substantially lower levels of life satisfaction, and nonresidential caregivers had significantly higher levels of life satisfaction, when compared with noncaregivers. Furthermore, whereas institutional caregivers also had higher levels of life satisfaction than noncaregivers, this difference is not significant. In-home caregivers, therefore, have lower levels of life satisfaction, whereas nonresidential caregivers are more satisfied with their lives than noncaregivers.

Model 2 introduces background controls. For depression, the difference between noncaregivers and in-home caregivers strengthened approximately 20%, whereas the remaining comparisons remained generally unchanged with the introduction of controls. For life satisfaction, the introduction of controls strengthened differences between noncaregivers and both in-home and nonresidential caregivers. The deficit in life satisfaction among in-home caregivers increased by almost 30%, while the advantage in life satisfaction among nonresidential caregivers increased by more than 40%. Adjusting for background factors associated with caregiving and psychological well-being therefore shows an important pattern—in-home caregivers experienced an excess of depression and deficit in life satisfaction, while nonresidential caregivers did not experience greater depression and were somewhat more satisfied with their lives than noncaregivers.

**Figure 2.**
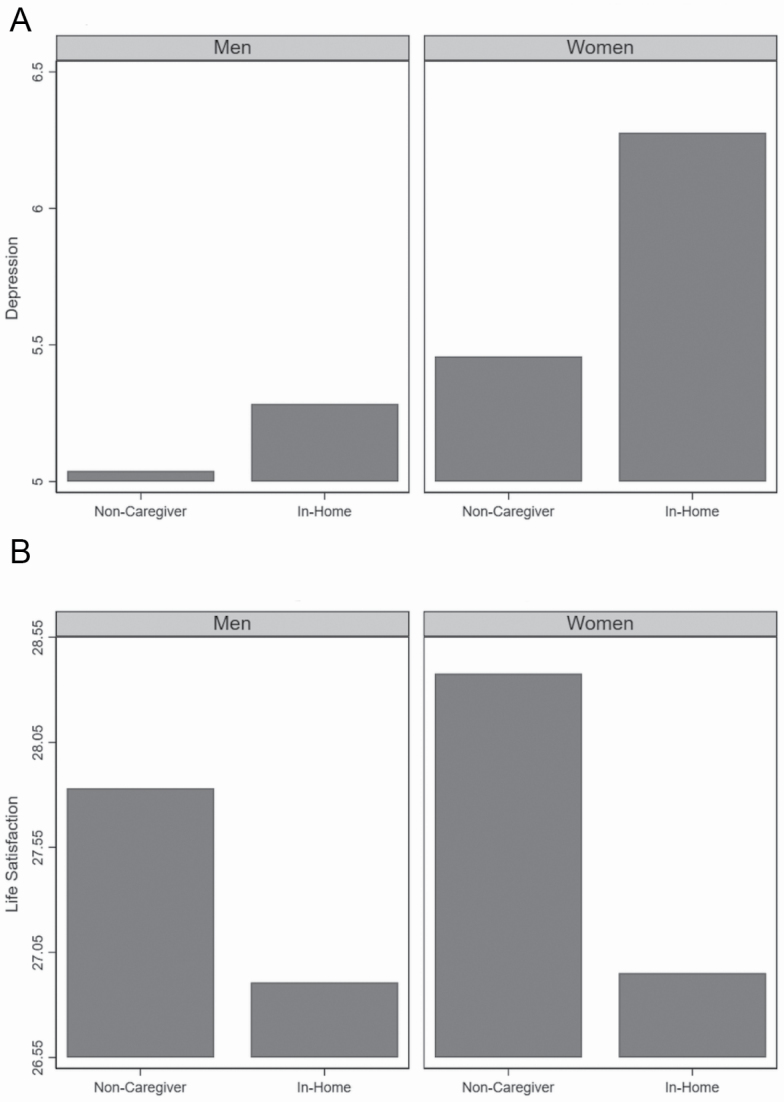
(**A**) Depression in noncaregivers and in-home caregiver—stratified by gender. (**B**) Life satisfaction in noncaregivers and in-home caregiver—stratified by gender.

The analyses to this point do not address whether these comparisons differ by gender. This question is addressed in Model 3, which tests interactions between gender and each location of caregiving. For both depression and life satisfaction, the interaction between in-home caregiving and gender is significant. These results indicate that gender differences in psychological well-being between caregivers and noncaregivers are evident, but only among in-home caregivers. Figure 2A illustrates this interaction for depression. Among both men and women, in-home caregivers had significantly higher levels of depression than noncaregivers, but the difference is stronger for women. A similar pattern can be seen for life satisfaction. In-home caregivers have significantly lower life satisfaction than noncaregivers, but the difference is larger for women.

## Discussion

Overall, these findings suggest that a dichotomous classification of caregiving status obscures the fact that there are both advantages and disadvantages for psychological well-being associated with caregiving which are circumscribed by the primary location of care. If we focused on caregivers only as a unitary category, we would conclude that caregivers tend to be more depressed than noncaregivers and that this difference is only partially explained by various controls. We would further conclude that the various background factors associated with caregiving obscure what is actually a deficit in life satisfaction among caregivers when compared with noncaregivers. Notably, these analyses would support a caregiving stress perspective, but not a caregiving rewards perspective. Yet, stratifying caregiving by the location of care shows a more complex pattern that provides support for both perspectives. When stratified by the location of care, only in-home caregivers reported greater depression and lower life satisfaction. Nonresidential caregivers did not differ significantly in depression from noncaregivers and reported higher life satisfaction. Institutional caregivers reported greater depression, but did not differ significantly from noncaregivers in terms of life satisfaction. These patterns were stronger among women than men.

That higher levels of depression and deficits in life satisfaction are evident primarily among in-home caregivers supports previous research indicating that caregivers face the greatest risk of negative psychological outcomes when they live in the same household as the care recipient (National Academies of Sciences, 2016). There are several reasons that this might be the case. In-home caregivers often provide care that is longer term, more extensive, and intensive. In addition, in-home caregivers also are likely to have more limited opportunities to take a break from caregiving compared to nonresidential community-based or institutional caregivers. There is a need for research that evaluates potential explanations for the increased risk of adverse psychological well-being specifically among in-home caregivers.

Our findings suggest that the main psychological well-being benefit of caregiving accrues to nonresidential caregivers of care recipients living in the community, as they reported higher life satisfaction than noncaregivers whereas institutional caregivers did not. The unique benefits of caregiving at a residence other than one’s own may well have to do with the intrinsic rewards of caregiving, such as providing a sense of fulfillment and meaning or increasing feelings of competency. Individuals providing in-home care are less able to appreciate the rewards of caregiving given the greater stress and demands associated with their roles. Institutional caregivers, on the other hand, may be more likely than in-home caregivers but less likely than nonresidential caregivers, to be in a position to experience the intrinsic rewards associated with caregiving. No significant differences in life satisfaction between institutional caregivers and noncaregivers may be the result of contradictions posed by a reduction in the demands of caregiving but increases in difficulties experienced on the other (e.g., dealing with institutional personnel, feelings of guilt). There is a need for research that focuses on the perceived rewards that may function as mechanisms through which caregiving activities enhance life satisfaction and/or other psychological well-being outcomes in different care contexts.

Our findings also point to the importance of considering how gender intersects with the location of care. If we had focused only on caregivers as a whole, we would not have identified a difference in psychological well-being outcomes between men and women. These differences become apparent only when caregivers are studied separately by the location in which care is provided, with the gender difference applying only to in-home caregivers. In-home caregivers are more depressed and less satisfied with life than noncaregivers, but these differences are stronger for women than men. This finding is in line with research suggesting that the negative mental health effects of providing care are greater for women (Heger, 2017). However, the finding that women’s greater vulnerability is limited to the in-home care context would appear to counter claims that women tend to be more responsive to caregiving and other stressors (Rosenfield & Mouzon, 2013), suggesting instead that it has much to do with the contexts within which caregivers are situated.

The current study has several limitations. First, because we drew on cross-sectional data, a causal link between caregiving and indicators of psychological well-being cannot be assumed. In addition, the data we used were restricted to those aged 45–85 years, thereby limiting the generalizability of our findings to caregivers within this age range. The differences in sample size across the three locations of care are also noted. Whereas in-home caregivers accounted for 23% of the caregiver sample and institutional caregivers accounted for just more than 10% of the caregiver sample, other nonresidential caregivers accounted for about 66%. The variable sample size among the groups might represent the national frequency given that the nationally representative data are used. Another limitation is that we only examined depression and life satisfaction as caregiving outcomes. Using additional measures of outcomes such as psychological distress or physical health might have provided a more holistic picture of caregivers’ well-being related to the location of care provided. However, we needed to select measures of both the positive and negative caregiving outcomes and thus, these other measures were not included in this study.

The findings of this study have important implications for policy and practice. In particular, they contribute to a growing body of evidence suggesting the need for tailored interventions that take into account the heterogeneity of caregiving (Young et al., 2020). This includes aspects of the caregiving context such as the location of the care provided as well as the gender of the caregiver. In-home caregivers in general, and women in-home caregivers in particular, are at heightened risk for reduced psychological well-being, suggesting the need for targeted interventions and support to be provided. Also, helping professionals such as social workers, clinicians, or health care workers need to use different interventions depending on whether they are working with in-home caregivers or nonresidential caregivers. Given that nonresidential caregivers actually benefit from providing care, interventions need to focus on identifying and bolstering positive aspects of the caregiving experience. Knowing that the location of care matters for caregivers’ psychological well-being would suggest that in-home caregivers might need more opportunities for taking a break from caregiving in a way that involves being physically as well as psychologically separated from the care site. Providing respite might help in-home caregivers alleviate their depressive symptoms. Interventions should be designed to both limit the stress and enhance the rewards of caregiving.

Overall, this study contributes to an enhanced theoretical understanding of caregiving experiences by demonstrating that the caregiving stress and caregiving rewards perspectives are both correct, but for different groups of caregivers. It is only by considering a more refined view of caregiving differentiated by intersections of gender and the location of care that these contrasting patterns become clear. Both the caregiving stress and caregiving rewards perspectives are relevant to the caregiving experience, but the location within which care is provided and the gender of the caregiver differentiate the relevance of each perspective. Thus, an integrated perspective that accommodates both the SPM and CEM is warranted. This approach would seek to incorporate both the stressors and rewards of caregiving, relating them to various outcomes, while also specifying the role of various contextual factors in defining when the stresses or rewards of caregiving may be more prevalent. Future research needs to further focus on what would bolster the positive experiences and reduce the negative ones.

## Funding

This work was supported by a Social Sciences and Humanities Research Council of Canada (SSHRC) grant (#430-2018-00437) to Y. Lee.
